# Evaluating A Patient-Led Health Literacy Program for People Living With Metastatic Breast Cancer

**DOI:** 10.1177/10732748261465064

**Published:** 2026-07-02

**Authors:** Brianna D. Taffe, Amy Beumer, Lesley Glenn, Maryam B. Lustberg, Mya L. Roberson

**Affiliations:** 1 41474 Department of Health Policy and Management, University of North Carolina at Chapel Hill Gillings School of Global Public Health, Chapel Hill, NC, USA; 2 Project Life, Central Point, OR, USA; 3 Division of Medical Oncology, Department of Internal Medicine, 156178 Yale School of Medicine, New Haven, CT, USA; 4 Center for Breast Cancer, 25045 Yale University Yale Cancer Center, New Haven, CT, USA; 5 169113 Lineberger Comprehensive Cancer Center, Chapel Hill, NC, USA

**Keywords:** metastatic breast cancer, health literacy, patient engagement, virtual intervention

## Abstract

**Introduction:**

Low cancer health literacy undermines patients’ ability to interpret complex information and participate in shared decision making, and is associated with worse outcomes. Evidence for effective health literacy interventions in metastatic breast cancer (MBC) remains limited. We evaluated a patient-led, virtual health literacy program designed to strengthen MBC-specific knowledge and self-efficacy.

**Methods:**

We conducted a mixed-methods, pre–post evaluation across two program iterations (Fall 2023; Spring 2024) delivered by a national, patient-led-MBC organization, Project Life. The five-week synchronous course called Spinning Science covered breast cancer subtyping, genetic/genomic testing, clinical trials, circulating tumor DNA, and information literacy, with small-group activities and polling. Program participants were adults living with MBC. De-identified pre/post surveys assessed (1) self-efficacy for engaging in health decisions, and (2) knowledge using items adapted from the Cancer Health Literacy Test aligned to course content. Paired two-sample t-tests examined pre–post changes (α=0.05). Open-ended responses were analyzed thematically.

**Results:**

Fifty-four people with MBC were enrolled (cohort 1, n=17; cohort 2, n=37); 46 provided matched pre– and post–surveys (14 and 32, respectively). Agreement with “I don’t know enough to make my own medical decisions” declined from 43% pre to 13% post (p<0.05), indicating improved self-efficacy. Baseline knowledge scores were high, and knowledge item gains were not statistically significant, consistent with ceiling effects. Post-program items showed >80% agreement for increased confidence in self-advocacy, improved health literacy, and sense of community. Qualitative feedback highlighted strengths like digestible content, approachable patient facilitators and flexible scheduling, as well as priorities for refinement such as continued access to materials, and more MBC-specific and numeracy content.

**Conclusions:**

A patient-developed, virtual health literacy program for people living with MBC showed meaningful improvements in self-efficacy, with actionable, participant-driven refinements between cohorts. This model offers a practical, scalable pathway for advancing self-efficacy within and beyond MBC.

## Plain Language Summary

People living with metastatic breast cancer (MBC) face complex choices about tests and treatments. We evaluated a five‑week, patient‑led online program created by Project Life by and for people with MBC to build practical health knowledge and confidence for decision‑making. The Spinning Science course covered tumor subtypes, genetic and genomic testing, clinical trials, circulating tumor DNA, and how to find and judge trustworthy information. The program used short talks, small‑group discussions, live Q&A, and polls to keep the material clear and actionable. Across two offerings (Fall 2023 and Spring 2024), 54 people enrolled and 46 completed both “before” and “after” surveys; the share who agreed with “I don’t know enough to make my own medical decisions” dropped from 43% to 13%, showing a meaningful gain in decision self‑efficacy. Because many participants began with high knowledge, we did not observe changes in pre- and post- health literacy. However, more than 80% of respondents reported increased confidence in self‑advocacy, improved health literacy, and a stronger sense of community by the end of the program. Participants praised approachable facilitators, digestible content, and flexible scheduling, and they asked for continued access to materials, a glossary, and more MBC‑specific and numeracy content (for example, help interpreting statistics and lab results). This evaluation has limits: participants were highly educated and familiar with MBC, we did not collect detailed demographics, and experience with MBC varied widely. These factors that can affect changes in knowledge and make it challenging to assess true program impact and equity. Overall, this patient-developed online synchronous MBC health literacy intervention improved confidence in health decisions for this cohort of individuals with MBC.

## Introduction

As cancer care becomes more personalized and complex, patients play an increasingly large role in shared healthcare decisions with multidisciplinary care teams, often requiring patient knowledge and activation.^1^Patient-centered educational programming is an essential tool for empowering patients to play a larger role in their care and improving their self-efficacy in making informed health decisions.^2-4^ Educational interventions can improve patient outcomes and self-efficacy for active participation in shared decision-making; however, low health literacy can compromise patients’ understanding of their disease and treatment, affecting their ability to make informed healthcare decisions.^5^

Though there is no single definition of health literacy, one definition is an individual’s capacity to obtain, understand, and act on basic information for health decision-making.^6^ Low health literacy can impede one’s ability to communicate with healthcare providers and participate in shared decision-making.^7^ Health literacy plays a critical role in disease control and prevention.^5,7-9^ Health literacy is closely intertwined with socioeconomic status, educational attainment, and other social determinants of health; as a result, the associations between health literacy and cancer outcomes often work through reinforcing correlative loops rather than independent pathways.^8,10,11^ Studies have found that women with lower health literacy, who are also more likely to experience socioeconomic disadvantage, tend to report poorer communication with their providers and higher levels of breast cancer-related decision regret compared to women with higher health literacy.^10^ Separately, lower health literacy has been associated with lower mammography screening rates.^12^ These findings collectively underscore the importance of health literacy as a modifiable factor within a broader constellation of social and structural determinants of cancer outcomes. For example, compared to women with high health literacy, women with low health literacy have poorer communication with their providers, higher levels of breast cancer decision regret, and lower mammography screening rates.^10,12^

Health literacy is also associated with cancer self-management behaviors like symptom management and medication adherence,^13^ and low health literacy can make it difficult for patients to understand complicated health information.^5-7^ Improving health literacy directly impacts medication adherence, utilization of preventive services, and active patient engagement in health decision-making.^14^ Beyond its associations with health behaviors and clinical outcomes, health literacy is closely linked to psychological well-being. Lower health literacy has been associated with greater cancer-related distress, uncertainty, and anxiety, as patients with limited health literacy may struggle to understand their diagnosis, interpret medical information, and engage meaningfully with their care teams.^5,7^ Improving health literacy and self-efficacy, therefore, has the potential to improve health behaviors and outcomes and also to support the emotional and psychological dimensions of living with cancer.

Information and efforts to improve health literacy are understudied unmet needs in metastatic breast cancer (MBC) patients globally, and in the United States.^15^ There are currently over 150,000 people living with MBC in the United States, a number projected to increase to over 169,000 in 2025.^16^ Though accurate patient-facing MBC information exists, the burden can fall to patients to search for MBC information online.^15^ Given the substantial technicality of medical literature, coupled with the proliferation of mis- and dis-information of cancer-related information, there is a great need for well-curated educational resources and tools to aid patients in shared decision-making.^17^

Health disparities can be reduced through intentionally designed, culturally competent patient education interventions.^18,19^ A considerable amount of patient education is delivered by nurses or patient navigators including social workers or community members, and can improve patient quality of life, knowledge of disease and disease-related resources, and treatment costs.^1^ However, nurses and navigators often have many other responsibilities and little time to assess health literacy, tailor patient education materials, and deliver interventions.^19^ Nursing literature supports using patient-led interventions to assess health literacy, improve patient engagement and program-related knowledge.^20^

Online delivery of patient education has proven effective in cancer populations,^21^ and there have been calls for partnership-based, needs-tailored patient empowerment interventions that are less resource-intensive on the healthcare system, for example, via web delivery, or patient-led interventions.^22,23^ Another critical element of these interventions is the opportunity for patients to revisit program materials once an intervention has ended to improve their self-efficacy and confidence in participating in shared decision-making.^24^ Few studies have aimed to evaluate and improve health literacy in MBC patients, and fewer have done so using participatory approaches. Therefore, our objective was to collaborate with Project Life, a virtual wellness community for people living with MBC to conduct a preliminary evaluation of Spinning Science, an MBC patient-led and delivered educational intervention designed to empower people living with MBC and improve their MBC-specific health literacy.

## Methods

### Community Partnership and Project Conceptualization

The community partner for this evaluation was Project Life, a patient-led virtual wellness organization serving individuals with metastatic breast cancer (MBC) and their loved ones across the United States. Project Life provides no-cost, patient-led virtual programming, including therapeutic art sessions, legal clinics, healing circles, and other supportive services.^25^ The senior author [MLR] and the Executive Director of Project Life [LG] established a collaborative relationship in 2022 through mutual cancer advocacy networks. During the initial phase of the partnership, the senior author [MLR] worked with the Executive Director [LG] and members of Project Life to conduct a qualitative needs assessment among organization members.^25,26^ Building on this foundational work, the Executive Director [LG[and a co-author [AB] approached the senior author [MLR] to conduct an evaluation of data collected during the initial iterations of Spinning Science, a five-week, synchronous online educational program designed to improve MBC-specific health literacy among participants.

### Program Setting and Description

Spinning Science was conceptualized by the Executive Director [LG] and developed by a co-author [AB], an MBC patient advocate with doctoral training in biology. Program objectives were reviewed by additional patient advocates, an oncologist co-author [MBL], and a researcher [MLR} to ensure relevance, accuracy, and accessibility. The program aimed to enhance participants’ confidence and knowledge to support informed self-advocacy and healthcare decision-making in the context of MBC treatment and survivorship care. A core program goal was to ensure accessibility for participants across a range of health literacy levels. The program was offered twice to members of Project Life: once in Fall 2023 (cohort 1) and again in Spring 2024 (cohort 2).

The program consisted of five sequential synchronous online sessions, each approximately 75 minutes in duration. Session topics and learning objectives were as follows: Session 1- Breast Cancer Subtyping (understanding receptor status, tumor biology, and implications for treatment); Session 2 -Genetic and Genomic Testing (distinguishing germline from somatic testing, BRCA and other relevant mutations); Session 3-Clinical Trials (trial phases, eligibility, informed consent, and patient rights); Session 4-Circulating Tumor DNA (ctDNA biology, liquid biopsy, and clinical applications); Session 5-Information Literacy (evaluating online sources, identifying misinformation, and navigating scientific literature). The program was facilitated by Project Life leadership, including a co-author [AB] with doctoral training in biology, higher education pedagogy, and lived experience of MBC. Between cohorts, refinements were made to address participant feedback from cohort 1, including expanded MBC-specific content, improved pacing, enhanced accessibility of materials, and the addition of a stand-alone session on reading scientific abstracts and posters. Each cohort was intentionally limited to fewer than 20 participants to facilitate interaction. Sessions incorporated small-group activities, live chat monitoring, polling, and opportunities for questions throughout to promote engagement and comprehension. Core learning objectives and survey instruments were held constant across both cohorts to preserve comparability of evaluation data.

### Participants, Recruitment, and Eligibility

Participants were adult members of Project Life living with MBC who self-selected into Spinning Science following organizational outreach. Members could only participate in the program once. There were no formal exclusion criteria. Participation in the program and completion of the evaluation surveys were voluntary.

### Data Collection and Measures

This study used a mixed-methods, pre–post program evaluation design to assess changes in health literacy–related knowledge, self-efficacy, and participant experiences associated with Spinning Science. Participants completed pre- and post-program surveys assessing changes in knowledge and self-confidence about engaging in health-related decisions, as well as open-ended questions soliciting feedback on program strengths and areas for improvement. Self-efficacy and knowledge were assessed using the Cancer Health Literacy Test tailored to the content most aligned with program learning objectives.^27^ Items used Likert-type response scales and were administered immediately before and after program participation. Six additional Likert-type items were administered at post-test only, as these items assessed program-specific outcomes not meaningful at baseline: (1) confidence in finding sources of health information since completing the program; (2) improved understanding of MBC; (3) confidence in self-advocacy; (4) confidence in advocating for others outside of oneself; (5) sense of community; and (6) comfort asking science-related questions. Qualitative data consisted of open-ended free-text responses to pre- and post-program survey questions. Open-ended survey items elicited narrative feedback on perceived benefits, clarity, and applicability, as well as suggestions for improving future iterations. This evaluation was determined to be exempt from human subjects review by the University of North Carolina at Chapel Hill (24-1721) and Advarra Institutional Review Boards (Pro00079056). All data shared with the evaluation team [BDT, MLR] were de-identified prior to analysis.

### Statistical Analysis

Following completion of the second program iteration, the authors met to establish roles and responsibilities for the evaluation. The senior author [MLR] and first author [BDT] were responsible for conducting quantitative and qualitative analyses of de-identified pre- and post-program survey data from both cohorts. Project Life retained ownership of the data. The senior author [MLR] and first author [BDT} received de-identified pre- and post-program survey data from Project Life for analysis. Quantitative analyses assessed changes in self-efficacy and knowledge-related survey items using paired two-sample *t*-tests. Statistical significance was defined as p < 0.05. The survey instrument was identical across cohort 1 and cohort 2; no items were added, removed, or reworded between program iterations. Paired two-sample t-tests assessed within-person pre-to-post changes in self-efficacy and knowledge items for which survey wording was identical at both time points. Two items for which question wording differed between the pre- and post-program surveys (addressing biomarker identification and ctDNA applications, respectively) were excluded from paired analyses to preserve item comparability. All t-tests reflect within-person comparisons and do not assume equivalence of program content across cohorts.

Qualitative data from open-ended survey responses were analyzed using thematic analysis to identify salient themes related to participant experiences, perceived benefits, and suggested improvements. Two authors [BDT, MLR] independently reviewed responses and collaboratively developed and refined thematic codes through an iterative process; discrepancies were resolved through discussion. Quantitative analyses were conducted using SAS version 9.4, and qualitative analyses were conducted using MAXQDA 2022 (version 22.8.0). Preliminary findings were presented to Project Life, where stratification of results by program cohort was recommended due to modifications made between program iterations. All quantitative and qualitative analyses were subsequently revised to account for cohort-specific differences.

## Results

### Analytic Results

In total, 54 people living with MBC participated in the Spinning Science program in cohort 1 (N=17) and cohort 2 (N=37). Across the cohorts, 14 of the 17 cohort 1 participants and 32 of the 37 cohort 2 participants responded to the pre- and post-program surveys, for 46 complete survey respondents. Across cohorts, in the pre-test, 43% (N=23) of participants reported that they agreed or strongly agreed with the statement “I don’t know enough to make my own medical decisions” compared to only 13% (N=6) who reported agreeing or strongly agreeing with that statement in the post-test (p<0.05), demonstrating significantly improved self-efficacy across both cohorts. In general, participants had high health literacy overall on pre-program surveys assessed using items from the Cancer Health Literacy Test. Consequently, there was no statistical difference between the pre- and post-surveys for the knowledge-related questions.

Qualitative analysis of free-text survey responses revealed that Spinning Science was responsive to participant feedback from cohort 1 to cohort 2 (Table 1). In the free-text responses to the pre-program survey, participants in cohort 2 were more focused on improving their MBC-specific knowledge or understanding than participants in cohort 1. While an equal number of cohort 1 participants reported wanting to improve their general science or cancer knowledge or understanding (N=6, 43%) and MBC-specific knowledge and understanding (N=6, 43%), more participants in cohort 2 reported wanting to improve their MBC-specific knowledge or understanding (N=20, 63%) than general science or cancer knowledge or understanding (N=16, 50%). Participants from both cohorts described the importance of websites and information that are fact-based, data-supported, easily readable and accessible, and from trusted sources (e.g., medical centers, pharmaceutical companies, and funding sources). Participants also looked for patient-advocate input, information based on personal experience, and recommendations as markers of website quality.

**Table 1.** Frequency of Qualitative Themes for Full Survey Respondents From Cohort 1 (N=14) and Cohort 2 (N=32)

Questions and themes	Cohort 1 (Fall 2023) N (%)*	Cohort 2 (Spring 2024) N (%)*
Pre-Survey		
1. What do you hope to gain from participating in this program? OR What are you most excited about learning?		
Improved general science or cancer knowledge	6 (43%)	16 (50%)
Improved MBC-specific knowledge	6 (43%)	20 (63%)
Treatment Information	5 (36%)	5 (16%)
2. What are you go-to site(s) for information about MBC? A maximum of three please. If you don’t have any that’s totally ok! Just write that.		
MBC-specific Websites	6 (43%)	9 (28%)
Google	3 (21%)	4 (13%)
Breast Cancer-Specific Websites	2 (14%)	12 (38%)
3. What makes a good website or health/science information source? In other words, how do you evaluate sources of information related to MBC?		
Supported by Data	4 (29%)	11 (34%)
Readable/Accessible	4 (29%)	1 (3%)
Reputable Sources or Authors	3 (21%)	3 (9%)
Post-Survey		
4. Are there any additional topics you think we should add?		
Reviewing Literature	2 (22%)	0 (0%)
Understanding Academic Posters	2 (22%)	0 (0%)
Understanding Lab Results	0 (0%)	1 (6%)
5. What did you enjoy about Spinning Science? This could be anything from the content (what we talked about) to how it was presented to logistics like time of day, Zoom meetings etc.		
Layout/Design	7 (54%)	10 (34%)
Presenters	6 (46%)	13 (45%)
Time (e.g., duration)	3 (23%)	9 (31%)
6. What could be improved about Spinning Science? This could be anything from the content (what we talked about) to how it was presented to logistics like time of day, Zoom meetings etc.		
More Time (e.g., for sessions, small group discussion)	5 (38%)	1 (4%)
Access to Materials After Program	3 (23%)	0 (%)
Pace of the Program	2 (15%)	1 (4%)

*Table 1 presents the frequency and percentage of participants endorsing each qualitative theme from open-ended survey responses by cohort. Because themes are not mutually exclusive and participants could endorse more than one theme, percentages sum to greater than 100% within questions. Given the non-independence of theme categories and small cell sizes (cohort 1, N=14), formal statistical testing of between-cohort theme frequencies was not performed.

**Figure 1.**
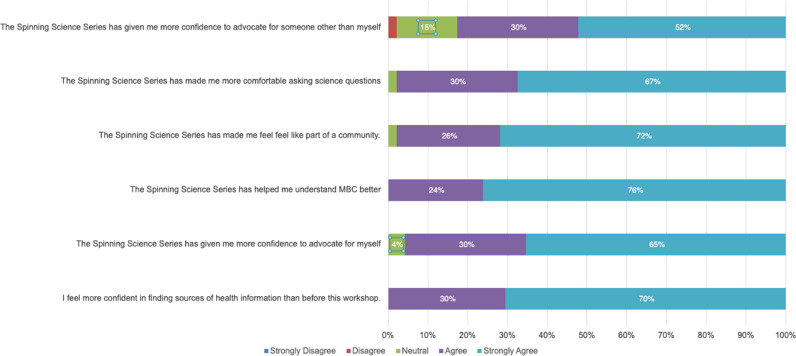
Proportion of program participants endorsing statements about the program in the post-test survey (N=46)

Several quantitative and qualitative items were asked only in the post-test. There was overall high agreement (>80%) for all items, including increased confidence in self-advocacy, health literacy, and feeling part of a community (Figure 1). In the post-program free-text responses, the most frequently suggested areas for improvement focused on timing, including pacing and duration of the program, with several participants wanting to spend more time on workshop sections, specifically in the first cohort. Cohort 1 participants also suggested improving accessibility of the handouts and workshop materials and adding a glossary of terms to the workshop binder. Some participants expressed interest in continued communication once Spinning Science concluded, and the ability to retake the course in the future.

In response to feedback from cohort 1, Project Life Leadership addressed participants’ desire to learn more about clinical trials and included a stand-alone workshop open to all Project Life members on how to read and understand academic posters, abstracts, and literature. The updated program also provided more MBC-specific content and addressed participant concerns about program pacing, workshop duration, and accessibility of program materials both during and after the program has finished. Participants appreciated how “digestible” the workshop information was, and the program’s built-in flexibility to attend morning or evening sessions depending on when they felt best or had treatment conflicts. Cohort 2 participants suggested future iterations invite a guest speaker, record the course sessions for later viewing, and include more breakout sessions to work in smaller groups. They also suggested adding content on disease progression, lab results, genetic and genomic testing, statistics, and MBC-relevant guidelines.

## Discussion

Overall, Spinning Science improved participating MBC patients’ self-efficacy and responded to the feedback and needs of the Project Life community by addressing areas for improvement from one iteration to the next. The academic-community partnership for this project provides an example of how to leverage empirical approaches to assess community-led programs.

MBC is a complex disease with increasingly complex testing and treatment modalities.^28^ This makes addressing health literacy in MBC patients crucial, since those with low health literacy may have difficulty accessing, understanding, and acting upon written and verbal health information, which also affects their ability to actively engage with their providers and the healthcare system.^7^ A scoping review on health literacy and cancer self-management behaviors found that low health literacy is associated with higher information needs, less information seeking behaviors, less uptake of screening behaviors and prescribed chemotherapy, and increased post-operative complications.^12^ While health literacy is correlated with socioeconomic status (SES) at the population level, SES does not fully determine an individual’s health literacy level.^29^ Systematic review evidence indicates that the relationship between health literacy and SES-related health disparities is inconsistent across outcomes^30^ and empirical mediation analyses suggest that SES only partially accounts health literacy variance.^29,31,32^ Specifically in the cancer context, in a population-based study of long-term breast cancer survivors, the majority of whom had high socioeconomic status, found that nearly 20% had marginal or inadequate health literacy and that SES and education together explained only a small proportion of the variance in health literacy scores.^33^ Moreover, general health literacy and cancer-specific health literacy are distinct constructs; patients with adequate general literacy may nonetheless have limited capacity to understand and act on the complex, rapidly evolving information encountered in cancer care.^11,34,35^ This distinction is particularly salient in MBC, where patients must navigate genomic testing, biomarker-directed treatment decisions, and clinical trial eligibility, domains in which even highly educated patients report significant knowledge gaps.^35^ A recent publication documented the importance of health literacy for cancer outcomes, as low health literacy was associated with higher all-cause mortality across all stages of breast cancer.^36^ This finding underscores the importance of improving health literacy not only to influence health behaviors among people with cancer but also to improve survival outcomes. Given the improving population-level survival for people with MBC due in large part to advancing therapeutic options, health literacy in this population specifically warrants particular focus to mitigate negative outcomes and inequities.^10,37^

While measures and papers on breast cancer health literacy and healthcare-directed education programs for health literacy delivered within clinical settings currently exist, to our knowledge, this is among the first patient-driven and led MBC health literacy initiative.^18,38^ A unique attribute of Spinning Science is that it was created for MBC patients engaged with Project Life by MBC patients in Project Life leadership. Participants were familiar with Project Life leadership and frequently commented on how much they appreciated leadership’s approachability throughout the course. Despite suggesting improvements, Spinning Science participants commented on how accessible the program was.

Barriers to health literacy in MBC extend beyond educational attainment and access to information. Disease-related factors, including the cognitive effects of treatment, fatigue, emotional distress, and the urgency of treatment decision-making, can impair patients’ ability to process, retain, and act on health information, even when that information is clearly presented.^5,7^ Social isolation, which may be exacerbated by the chronicity of MBC and the relative rarity of metastatic-specific peer communities, further limits patients’ access to trusted information sources and informal support networks. The virtual, community-based design of Spinning Science was explicitly intended to address several of these barriers, offering flexible scheduling, peer facilitation, and a supportive learning community, and participant feedback suggests that these structural features were perceived as meaningful facilitators of engagement.

The virtual delivery of Spinning Science offered several meaningful advantages. Synchronous online sessions enabled participation regardless of geographic location, treatment schedules, or physical health limitations factors that are particularly salient for people living with MBC who may face significant logistical barriers to in-person engagement. Participants particularly appreciated the flexibility to attend morning or evening sessions, depending on treatment schedules and personal well-being. Virtual delivery also reduced the resource burden on the community organization and enabled rapid scalability to future cohorts. At the same time, virtual delivery presents challenges, including digital literacy requirements, disparities in technology access, and the potential for reduced interpersonal connection compared with in-person programming. Participants noted a desire for recorded sessions and continued access to materials, suggesting the need for asynchronous components to complement synchronous programming. Future iterations of the program should consider hybrid or fully asynchronous delivery options to reach individuals with limited access to technology or irregular availability.

Across recent reviews, the evidence base for cancer health literacy interventions has expanded but remains uneven in maturity and scope.^11,35,39,40^ Mixed-studies syntheses confirm that low health literacy is consistently associated with difficulties processing cancer information, poorer experiences of care, and lower quality of life, reinforcing the clinical rationale for intervening beyond screening alone.^11^ Importantly, systematic reviews have demonstrated that most evaluated interventions still center on patient-level education and communication skills, with many studies in formative or pilot phases and comparatively few rigorous randomized trials demonstrating downstream clinical impact.^39^ The present study does not address this gap directly; rather, it contributes formative, preliminary evidence from a community-partnered, patient-led program evaluation. As such, it offers an empirical foundation and a practical model on which future, more rigorous evaluations can be designed and built. Findings from the initial development, implementation, and evaluation of Spinning Science yielded several lessons relevant to community-academic partnerships seeking to promote health literacy in oncology. These lessons informed subsequent program iterations and have broader implications for the design and evaluation of patient-centered educational interventions. Substantive reflection on program content identified health numeracy as an important area for future enhancement. Although the program addressed multiple domains of cancer-related health literacy, project team discussions identified limited emphasis on quantitative reasoning, despite the central role of statistics and probability in MBC prognostication and treatment decision-making. This finding suggests that health numeracy represents a critical, and often under-addressed, component of oncology health literacy that warrants intentional integration into future educational interventions^41^.

Early program evaluation underscored the importance of methodologically robust survey design to support meaningful assessment of health literacy-related outcomes. Feedback from the first two program iterations highlighted limitations in the initial evaluation instruments, prompting collaboration with the academic partner to strengthen survey design and analytic rigor. For future program iterations, demographic questions were expanded to better characterize participants, and content knowledge items were standardized to multiple-choice formats with a single correct response. These changes in assessment will improve the interpretability of results and enhance the program’s ability to detect changes in knowledge across future cohorts.

This evaluation has several limitations. First, the study population had high baseline health literacy and education levels, as well as substantial familiarity with MBC which likely reduced our ability to detect meaningful pre-post gains in knowledge creating a ceiling effect. Second, because this evaluation was implemented by a patient-led community organization rather than an academic institution, no demographic or clinical data were collected, including age, time since MBC diagnosis, treatment status, functional status, or sociodemographic characteristics. This precludes any examination of subgroup differences in outcomes and limits our ability to characterize the study population. Future iterations should prospectively collect these variables to enable subgroup analyses and to assess whether program impact varies by disease duration, treatment phase, or other patient-level factors. Third, the cohort was heterogeneous in MBC experience, some members have lived with the disease for a decade or more while others were more recently diagnosed, a factor that could influence baseline MBC knowledge when entering the program. Looking ahead, future iterations should intentionally reach and retain individuals with lower baseline health literacy and self-efficacy to more fully evaluate impact and equity. Notwithstanding these limitations, this patient-led model provides a critical assessment of the general information needs of people with MBC and demonstrates that well-designed, general cancer health-literacy content can be adaptable and applicable to this population. The work offers a practical foundation on which to build more rigorous, inclusive, and scalable programs.

## Conclusions

Overall, Spinning Science a patient-developed online synchronous MBC health literacy intervention improved confidence in health decisions for this cohort of individuals with MBC. Given the increasing complexity of treatment regimens over time there is great urgency for developing tools to aid patients in understanding their cancer care.

## Data Availability

The data that support the findings of this study are not publicly available due to the information they contain about people living with MBC. Access to the data may be granted upon completion of all required data use agreements and approval from Project Life upon reasonable request. Interested researchers should contact the corresponding author for further information.*

## Appendix

### Abbreviations

ctDNA: Circulating tumor deoxyribonucleic acid
MBC: Metastatic Breast Cancer
SES: Socioeconomic status.
